# Potential feeding deterrents of *Adelges tsugae* found in biological control flies

**DOI:** 10.1007/s00114-025-01996-y

**Published:** 2025-06-02

**Authors:** Olivia Andrews, Anne C. Jones, Mark Whitmore, Scott Salom

**Affiliations:** 1 Department of Entomology, Virginia Tech, Blacksburg, VA 24061 USA; 2 Department of Natural Resources and the Environment, Cornell University, Ithaca, NY 14853 USA

**Keywords:** *Leucotaraxis argenticollis*, *Tsuga canadensis*, Biocontrol, Anthraquinones, Invasive species

## Abstract

The invasive hemlock woolly adelgid, *Adelges tsugae* (HWA, Hemiptera: Adelgidae), is a detrimental pest to native eastern and Carolina hemlocks. In the last 2 decades, biological control utilizing two species of *Laricobius* beetles (Coleoptera: Derodontidae) has been of focus in the widespread effort to control HWA. Recently, two species of silver flies, native to the Pacific Northwest, *Leucotaraxis argenticollis* Zetterstedt and *Leucotaraxis piniperda* Malloch (Diptera: Chamaemyiidae), are being investigated as additional biological control agents. Releasing these two silver fly species in the eastern United States has yet to result in the establishment of these predators. During laboratory studies, *Leucotaraxis* larvae excreted a black substance in response to being disturbed, which contained anthraquinones previously detected in HWA. Previous research on *Laricobius* spp. found that the beetles likely sequestered these compounds from HWA. These compounds are feeding deterrents in other insect species. *Leucotaraxis argenticollis* life stages, their excrement, and honeydew produced by HWA were collected and analyzed by gas chromatography–mass spectrometry. Results showed that these anthraquinones were detected in various life stages of *Le. argenticollis* immatures and adults that fed on HWA. They were not detected in *Le. argenticollis* adults that were only fed artificial diet and water, indicating that *Le. argenticollis* sequesters the anthraquinones from HWA and may transmit the compounds to their eggs.

## Introduction

Since its discovery in 1951 in Virginia, the hemlock woolly adelgid (HWA), *Adelges tsugae* Annand (Hemiptera: Adelgidae), has killed eastern hemlock (*Tsuga canadensis* (L.) Carr.) and Carolina hemlock (*Tsuga caroliniana* Engelm) throughout much of their native range in the eastern United States (U.S.) (Ellison et al. 2018). *Adelges tsugae* feeding at high densities decreases tree health, resulting in needle drop that limits new shoot growth. Tree mortality occurs in as little as 4 years after initial infestation (McClure 1991).

For 2 decades, the main biological control agents of *A. tsugae* have been two beetle species in the family Derodontidae, *Laricobius nigrinus* Fender and *L. osakensis* Shiyake and Montgomery. Following their release and establishment in the eastern U.S. (Mausel et al. 2010), these beetles impact the first generation of *A. tsugae* (Jubb et al. 2020). However, another study demonstrated that the second generation of *A. tsugae* populations was able to recover regardless of the impact on the first generation (Crandall et al. 2020). This is likely due to *Laricobius* spp. completing pupal development in the soil, resulting in an absence of predators for the second generation of *A. tsugae*. In the Pacific Northwest, the second most abundant predators of *A. tsugae* were two species of silver flies in the family Chamaemyiidae, *Leucotaraxis argenticollis* Zetterstedt and *Leucotaraxis piniperda* Malloch (Kohler et al. 2008). In their native range, *Leucotaraxis* spp. are active after *Laricobius* spp. pupate in the soil (Dietschler et al. 2021). Investigations on *Leucotaraxis* spp. establishment, efficacy in reducing *A. tsugae* populations, and life histories are ongoing (Preston et al. 2023).

Since *A. tsugae* are non-mobile in most life stages, they secrete a wax covering and produce an anthraquinone, chrysophanol (1, 8-dihydroxy-3-methylanthraquinone) and an anthrone, chrysarobin (1, 8-dihydroxy-3-methylanthrone) (Skinner et al. 2003; Jones et al. 2012, 2016), which have feeding deterrent properties in some chrysomelid beetles (Hilker and Schulz 1991 and Howard et al. 1982). *La. nigrinus* beetles appear to sequester chrysarobin and chrysophanol from their *A. tsugae* prey, possibly investing these compounds in maternal protection for their eggs (Jones et al. 2014). As part of our work to investigate the life history of *Le. argenticollis* and their interaction with *A. tsugae* prey, we examined all life stages of this fly to understand their interaction with adelgid-derived anthraquinones.

## Materials and methods

### Collection of material

*Le. argenticollis* adults from HWA-infested western hemlock, *Tsuga heterophylla* (Raf.) Sarg., collected across 13 sites in western Washington and shipped to Virginia Tech’s Beneficial Insect Containment Facility (BICF) were used for this study. Adult flies were stored in 3.79 L holding containers at 15 °C:10 °C (L:D) and a photoperiod of 14:10 (L:D). Adults were fed an artificial diet of wheast (1:1 ratio of whey/yeast mixture and water) (Beneficial Insectary Inc.) and water until sexually mature. Adults were added to containers holding HWA-infested eastern hemlock to rear *Le. argenticollis* larvae. The following life stages were collected for analysis: adults that fed on wheast, adults that fed on HWA honeydew, eggs, early instar larvae, late instar/pre-puparium, and puparium. Honeydew produced by HWA and excrement of *Le. argenticollis* larvae were also collected with forceps which were cleaned before each collection. Approximately 10 individuals for each life stage of *Le. argenticollis* were placed in a few drops of methanol in a vial with a Teflon-lined cap for analysis.

### Chemical analysis

To detect chrysarobin and chrysophanol, pooled samples of each life stage were analyzed by gas chromatography–mass spectrometry (GC–MS) performed in the EI mode using a Shimadzu QP 2020 GC/MS equipped with a RTX-5, 30 m × 0.25 mm i.d., column (Restek Corporation, 110 Benner Circle, Bellefonte, PA 16823). The instrument was programmed from 60 to 250 °C at 5°/min, from 60 to 250 °C at 10°/min, or from 60 to 280 °C at 20°/min and held at 280 °C for 30 min. Mass spectra and gas chromatographic retention times were identical to standards from Jones et al. (2012, 2014). Briefly, the mass spectra of two aromatic components, *m/z* = 254 (100) and *m/z* = 240 (100), matched those of chrysophanol and the corresponding anthrone, chrysarobin. The ratios of the two compounds were determined from the peak areas derived from their intense parent ions using the instrumental software.

## Results

**Fig. 1:**
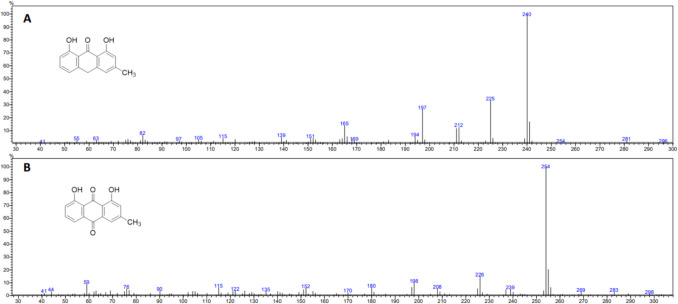
The structures and mass spectra of **A** chrysarobin (1, 8-dihydroxy-3-methylanthrone) and **B** chrysophanol (1, 8-dihydroxy-3-methylanthraquinone) from *Leucotaraxis argenticollis* silver flies

The parent ion of chrysarobin and chrysophanol is the base peak of these compounds and was detected by a standard fragment ion search. The compounds were detected in *Le. argenticollis* (Fig. 1) adults and in early instar larvae at low levels, but not in late instar larvae. Both compounds were detected in trace amounts in the puparium. Interestingly, these compounds were detected in *Le. argenticollis* adults that fed on honeydew from HWA but not in wheast-fed adults. These compounds were also detected in excretion produced by the *Le. argenticollis* larvae (Table 1).

**Table 1:** Ratio of chrysarobin and chrysophanol in life stages of *Leucotaraxis argenticollis*. Adults fed on artificial diet and *Adelges tsugae* honeydew and the honeydew alone are indicated

*L. argenticollis* life stage	# individuals	Chrysarobin:chrysophanol
Egg	30	5:1
Early instar larvae	10	1.5:1 (below baseline)
Larvae (secretions)	n/a	10:1
Pre-pupal larvae	10	Nd:nd
Puparia	10	2 (trace):1 (trace)
Adult (wheast)	10	Nd:nd
Adult (HWA honeydew)	10	2.5:1
HWA honeydew	n/a	3.5:1

## Discussion

Here we report the horizontal and vertical transmission of chrysarobin and chrysophanol from prey to predator and from mother to offspring of new biological control agents for an important invasive pest. Chrysarobin and chrysophanol were detected in the honeydew excreted by HWA and in adult *Le. argenticollis* feeding on anthraquinone-containing honeydew from HWA but were not detected in wheast-fed *Le. argenticollis*. This strongly suggests that chrysarobin and chrysophanol are horizontally transferred from the HWA honeydew excretions to the adult predator, *Le. argenticollis*. Chrysarobin and chrysophanol were also detected in *Le. argenticollis* fly larvae and pupae as they were with *Laricobius* spp. (Jones et al. 2012). The larvae of both *Laricobius* spp. beetles and *Le. argenticollis* flies feed on HWA eggs, which contain these compounds. Additionally, our results suggest that the compounds are being vertically transferred from adult *Le. argenticollis* to eggs as they were detected in eggs laid by honeydew-fed *Le. argenticollis* females.

Anthraquinones are antifeedants in several insects; however, to date, these compounds are not sequestered from food (a host plant or prey insect) and are likely biosynthesized de novo (Eisner et al. 1980; Howard et al. 1982; Hilker and Schulz 1991; Jones et al. 2012). The anthraquinone-containing tansy leaf beetle larvae were significantly less palatable to bird predators compared to mealworms offered as control (Hilker and Köpf 1994). Additionally, these compounds in the elm leaf beetle and cochineal bugs have feeding deterrent activity against ants (Howard et al. 1982; Hilker and Schulz 1991).

The detection of chrysarobin and chrysophanol in HWA honeydew-fed *Le. argenticollis*, but not wheast-fed flies, strongly suggests that the anthraquinones detected in *Le. argenticollis* are obtained from HWA. Female wheast-fed *Le. argenticollis* have not successfully laid eggs in captivity (personal observation), but eggs from HWA honeydew-fed *Le. argenticollis* contain chrysarobin and chrysophanol, likely incorporated by the female into the eggs. In leaf beetles, anthraquinones are also likely invested in eggs by the female who produces these compounds via enzymes (Pankewitz and Hilker 2006; Pankewitz et al. 2007). In the *Le. argenticollis*–HWA system, the anthraquinones are likely sequestered from HWA honeydew by the adults and transferred to eggs.

It is unclear if the anthraquinones in *Le. argenticollis* eggs are contributed from male or female flies or if they are sequestered during larval feeding on HWA or adult feeding on HWA honeydew (both of which contain chrysarobin and chrysophanol). Due to the trace and undetectable levels of these compounds in *Le. argenticollis* pre-pupal larvae and puparia, the compounds may be derived from one or both parents (Table 1). Further work will elucidate the maternal and paternal investment of chrysarobin and chrysophanol through feeding/rearing assays and additional GC–MS analysis.

*Le. argenticollis* fly larvae feed on HWA eggs, which contain chrysarobin and chrysophanol, as their main food source (Jones et al. 2012; 2014). The change in detectability of these compounds in *Le. argenticollis* immatures as they progress through instars can be explained by the larvae feeding on their HWA food source (Table 1). Furthermore, *Le. argenticollis* larvae produce an excrement when prodded with a paint brush (personal observation). Defensive regurgitation has been reported in some lepidopteran and coleopteran larvae (Freitas and Oliveira 1992). Chrysarobin and chrysophanol were detected in *Le. argenticollis* larval excretions, indicating that they may utilize the anthraquinones for defense in the larval stage. The fly puparia are also anchored to hemlock branchlets with a visually similar excretion (personal observation). The presence of chrysarobin and chrysophanol in this attachment may deter predators from disturbing the puparia. The antimicrobial nature of these compounds may also provide an additional layer of pathogen protection to the vulnerable pupal stage (Hilker et al. 1992).

The findings demonstrate a unique example of antifeedant sequestration from prey by a dipteran predator in the context of invasive species biology. Understanding the ability of specialist HWA predators to not only sequester these compounds but also contribute them on to the subsequent generation allows us to narrow our search for biological control agents of the invasive HWA. The ability of these specialized predators of HWA to sequester anthraquinones demonstrates their close association with each other and should be used when evaluating other potential biological control agents in this system. Future work will include quantification of these compounds in all the life stages of *Le. argenticollis*, the degree of their investment to offspring, and establish the deterrent effect of these compounds on more generalist predatory insects.

## Data Availability

No datasets were generated or analysed during the current study.
